# Antimicrobial resistance and prevalence of tetracycline resistance genes in *Escherichia coli* isolated from lesions of colibacillosis in broiler chickens in Sistan, Iran

**DOI:** 10.1186/s12917-020-02488-z

**Published:** 2020-08-03

**Authors:** Mohammad Jahantigh, Keyvan Samadi, Reza Esmaeelzadeh Dizaji, Saeed Salari

**Affiliations:** 1 Department of Clinical Sciences, School of Veterinary Medicine, University of Zabol, Zabol, Iran; 2 Department of Clinical Sciences, School of Veterinary Medicine, University of Tehran, Tehran, Iran; 3 Department of Pathobiology, School of Veterinary Medicine, University of Zabol, Zabol, Iran

**Keywords:** Broiler chickens, Colibacillosis, *E. coli*, Resistance genes, Prevalence

## Abstract

**Background:**

Antibiotics have long been the first line of defense to prevent *Escherichia coli* infections, but they have lost their potency since bacteria have grown increasingly resistant to treatment. The present research aimed to study the drug resistance and the prevalence of tetracycline resistance genes in *E. coli* isolated from broilers with colibacillosis.

**Results:**

The results showed that the most prevalent type of drug resistance was to tetracycline at 95.0%, and the least was to gentamicin at 21.7%. The prevalences of antimicrobial resistance among the tested antibiotics were significantly different (*p* < 0.001). A statistically significant difference was observed between the prevalence of the *tet* genes (*p* < 0.001). The *tet*D positive isolates and antibiotic sensitivity to tetracycline showed statistical significant differences (*p* = 0.017).

**Conclusions:**

Considering the results, *tet*A is the most common tetracycline resistance gene, and the presence of *tet*D and antibiotic sensitivity to tetracycline had a significant relationship in *E. coli* isolated from colibacillosis infections.

## Background

Antibiotic are mostly used in animals, including chickens, for the treatment or prevention of diseases [1–3]. Some of these agents, such as avilamycin, avoparcin, flavomycin, monensin, and salinomycin, have also been used in a few countries to increase chick growth rates [1]. The high consumption of antibiotics by livestock is a global problem that can increase the antibiotic resistance of human and animal bacteria such as *Escherichia coli* [4–6]. Antibiotic resistance in poultry bacteria and transfer of this acquired ability to human bacteria can disrupt the treatment of human infections [4].

Tetracyclines are broad-spectrum antibiotics that are widely used against gram-negative and gram-positive bacteria. These drugs prevent the binding of aminoacyl-tRNA to the 30s ribosomal subunit and disrupt protein synthesis, which prohibits the growth of sensitive bacteria [7]. Because of the numerous advantages of tetracyclines, such as widespread availability, low cost, and few side effects, the use of these kinds of antibiotics for the treatment of animal and human infections has been increasing in recent years [8]. This has led to the emergence of tetracycline-resistant bacteria, which is now limiting the use of these antibiotics [9].

Tetracycline resistance genes are generally coded in plasmids and transposons and are transmitted through conjugation. However, the relevant genes are also found in the chromosome in some isolates [10, 11]. Mechanisms of resistance to tetracycline through the acquisition of *tet* genes mainly include efflux pumps, ribosomal protection, and enzymatic deactivation. Mutations also contribute to the antibiotic resistance [12]. The *tet* genes found at the highest frequency in gram-negative bacteria are related to efflux pumps, which are coded by the *tet*A, *tet*B, *tet*C, *tet*D, and *tet*G genes [13, 14].

Resistance to tetracycline can be used for evaluating antibiotics resistance genes. Also, the epidemiological-molecular evaluation of resistant strains to these compounds can broaden the knowledge of appropriate treatments and prevent loss of capital [15]. Strategies to prevent the spread of antibiotic resistance require surveillance of its encoding genes. This study provides new insight for explaining the combination tetracycline-resistant encoding genes that may synergistically enhance the antimicrobial resistance against tetracycline in *E. coli* isolates.

Hence, the present research aimed to study the resistance to antimicrobial agents and the prevalence of tetracycline resistance genes (*tet*A, *tet*B, *tet*C, and *tet*D) in *E. coli* isolated from broiler chickens affected with colibacillosis on farms in Sistan, Sistan and Baluchestan Province, Iran.

## Results

**Fig. 1:**
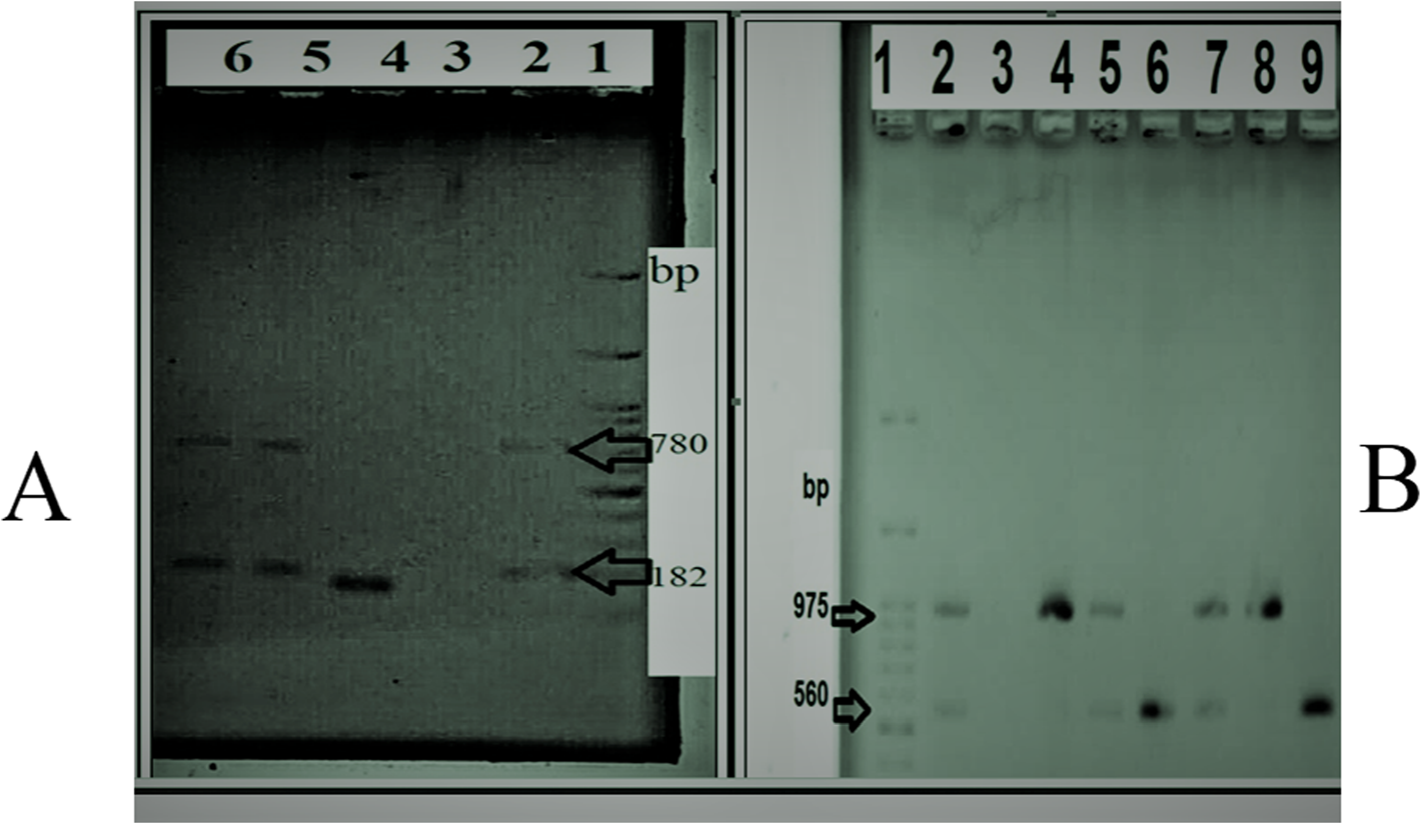
**a:** Electrophoresis results for detecting the *tet*A and *tet*D genes. Lanes: 1, marker; 2, positive control; 3, negative control; 4, isolate contains *tet*A gene; 5 & 6, isolates contain *tet*A and *tet*D genes. **b:** Electrophoresis results for detecting the *tet*B and *tet*C genes. Lanes: 1, marker; 2, positive control; 3, negative control; 4 & 8, isolates contain *tet*B gene; 6 & 9, isolates contain *tet*C gene; 5 & 7, isolates contain *tet*B and *tet*C genes

The antimicrobial drug susceptibility patterns of the *E. coli* isolates are presented in Table 1. The frequency of resistance to tetracycline, ciprofloxacin, co-trimoxazole, lincospectin, cefuroxime, and gentamicin were 95.0, 88.3, 86.7, 53.3, 46.7, and 21.7%, respectively (Supplementary materials). A total of 52/60 (86.6%) isolates were identified as multidrug resistance strains. The prevalence of antimicrobial resistance among the tested antibiotics was significantly different (*p* < 0.001). The gel photograph of the amplified products is presented in Fig. 1; Additional file 1: Fig. 1. Antibiotic sensitivity to tetracycline dependent on the presence or absence of the *tet*A, *tet*B, *tet*C, and *tet*D genes is shown in Table 2. The *tet*D positive isolates and antibiotic sensitivity to tetracycline showed statistical significant differences (*p* = 0.017). There was no observed statistical association between *tet* genes and other antibiotics sensitivity, including to gentamicin, lincospectin, cefuroxime, ciprofloxacin and co-trimoxazole (statistical results not shown). The prevalence of the *tet*A, *tet*B, *tet*C and *tet*D genes was 96.7, 38.3, 31.7, and 8.3%, respectively. A statistically significant difference was observed between the prevalence of the *tet* genes (*p* < 0.001). The results showed that all isolates had at least one of the tetracycline resistance genes. The distribution of *E. coli* isolates harboring one, two, three, and four *tet* genes were 43.3, 43.3, 13.3, and 0.0%, respectively.

**Table 1:** Antimicrobial drug susceptibility patterns of the *Escherichia coli* isolates

	Resistant (%)	Intermediate (%)	Susceptible (%)
**gentamicin (10 μg)**	21.7	0.0	78.3
**lincospectin (15/200 μg)**	53.3	8.3	38.3
**cefuroxime (30 μg)**	46.7	35.0	18.3
**ciprofloxacin (5 μg)**	88.3	1.7	10.0
**co-trimoxazole (1.25/23.75 μg)**	86.7	0.0	13.3
**tetracycline (30 μg)**	95.0	1.7	3.3

**Table 2:** Antimicrobial susceptibility to TE_30_ depending on the presence or absence of the *tet*A, *tet*B, *tet*C, and *tet*D genes, among 60 isolates of *Escherichia coli*

Antimicrobial agent	Susceptibility	***tet***A	***tet***B	***tet***C	***tet***D
**TE**_**30**_	Resistant	56 (97%)	22 (96%)	19 (100%)	3 (60%)
	Intermediate	1 (2%)	0 (0%)	0 (0%)	1 (20%)
	Susceptible	1 (2%)	1 (4%)	0 (0%)	1 (20%)
**Statistical association between TE**_**30**_**&*tet*(s)**		**0.096**	**0.583**	**0.308**	**0.017**^a^
**Number of isolates with*tet*genes**		**58**	**23**	**19**	**5**

TE_30_: tetracycline

^a^Significant

## Discussion

Tetracycline is the most frequently used antibiotic in the treatment and control of various poultry diseases. Tetracycline can be administered orally and has only a very low risk of major side effects. Furthermore, tetracycline is one of the cheapest antimicrobial agents available [16, 17]. In this study, resistance to more than one antimicrobial agent was observed for many of the *E. coli* isolates (52/60; 88.6%), and 95.0% of the isolates showed tetracycline resistance.

In another study, the frequency of tetracycline resistance of *E. coli* isolated from colibacillosis in Iran was reported at 96.0% [5]. Besides, the antimicrobial resistance prevalence of *E. coli* isolated from turkey in Iran, for gentamicin, tetracycline, co-trimoxazole, norfloxacin and cefuroxime, were 5, 51.7, 23.3, 5 and 48.3%, respectively [18]. Adesiyun et al. (2007) reported similar levels of resistance to antimicrobial agents among isolates of *E. coli* to tetracycline (58.5%), enrofloxacin (25.4%), gentamicin (9.3%), and sulphamethoxazole/trimethoprim (15.3%) [19]. The excessive use of tetracycline compounds for the treatment of poultry diseases could be the reason for their high rate of resistance to this type of antibiotic [5, 20–23].

Fluroquinolone compounds like ciprofloxacine are critically important antimicrobials for treating severe infections in human and reduced susceptibility to quinolone can lead to treatment failures and is considered a public health risk [24]. According to the results of the current study, the frequency of tetracycline resistance (95.0%) was only slightly higher than the frequency of quinolone resistance (88.3%).

In this study, we observed that 43.30 and 13.3% of the *E. coli* isolates harbored two and three tetracycline resistance genes, respectively. The presence of multiple tetracycline resistance genes in *E. coli* has been previously reported by Sandalli et al. (2010). They detected that 1.9% of *E. coli* isolates harbored both the *tet*A and *tet*B genes. They observed that the *tet*A and *tet*B genes could be well expressed in *E. coli* and subsequently impart resistance to tetracycline [25]. Furthermore, Koo and Woo (2011) also observed that 1.6% of *E. coli* isolates had both *tet*A and *tet*B simultaneously [12].

According to the results of our research, *tet*D showed a significant relationship with antibiotic sensitivity to tetracycline. This correlation may be due to the influence of the environment, different loci, mRNA copy numbers, different processes at the transcriptional/translational levels, and the expression patterns of the genes. However, Chopra and Roberts (2001) have reported that *tet*B provides additional resistance against doxycycline, while *tet*A induces resistance against tetracycline, oxytetracycline, and chlortetracycline [16].

In the current study, all isolates of *E. coli* had at least one *tet* gene, and *tet*A (96.7%) and *tet*B (38.3%) were observed at the highest frequencies. Similar to our results, in a study of meat and meat products, Koo and Woo (2011) observed that most of the isolates (98.3%) had at least one *tet* gene, with *tet*A (52.4%) and *tet*B (41.3%) showing the highest frequencies while the lowest frequencies were found for *tet*C (1.7%) and *tet*D (0.8%) [12]. Guerra et al. (2003) reported the prevalences of *tet*A and *tet*B were 66 and 42%, respectively, in *E. coli* isolated from cattle, swine, and poultry [26]. Maynard et al. (2004), in a study on *E. coli* isolated from human and animal samples, showed that the frequency of *tet*A was greater than *tet*B in isolates from both sources [27]. Seifi and Khoshbakht (2016) reported that 73% of *E. coli* strains isolated from Iranian broiler flocks were tetracycline resistant. Moreover, 46 and 41% of the isolates contained *tet*A and *tet*B genes, respectively [28].

## Conclusions

The evaluation of the tetracycline resistance pattern can be helpful in choosing proper antibiotic agents for the treatment of poultry diseases. According to our results, *tet*A is the most common tetracycline resistance gene, and the presence of *tet*D and antibiotic sensitivity to tetracycline has significant relationships in *E. coli* isolated from colibacillosis. By identifying the types of genes responsible for resistance, more effective approaches may be developed to treat infectious diseases. The results of this study emphasize the need for cautious use of tetracycline in poultry production to decrease the prevalence of tetracycline-resistant *E. coli*. However, it is also recommended that antibiotics from different classes will need to be used to reduce antimicrobial resistance, and more efficiently treat infectious diseases of poultry.

## Methods

### Sample collection and bacterial isolation

The study population consisted of commercial broilers affected by colibacillosis. Sampling was conducted on eight broiler farms during 2017 to 2018 in Sistan, Iran. Verbal informed consent was obtained from the all farm owners for sample collection. This study was approved by the Ethics Committee of the University of Zabol with the approval number IR.UOZ.REC.1395.11.

After necropsy and identification of the respective lesions, the samples were taken from the infected chicks. Lesions were sampled by using a sterile swab and then the swab was placed in 5 ml of tryptic soy broth (TSB) medium and transferred to the microbiology laboratory of the Faculty of Veterinary Medicine of Zabol University. The TSB media were incubated at 37 °*C* for 24 h and then sub-cultured on MacConkey agar and eosin methylene blue (EMB) agar media (Merck, Germany).

A total of 60 isolates of *E. coli* were procured from the colibacillosis-related lesions and identified using precise microbiological and biochemical methods. In summary, bacterial isolates with typical colony morphology on MacConkey and EMB agar media that were lactose, indole, and methyl red positive, while Voges–Proskauer, citrate, urease, and H_2_S negative, were considered as *E. coli* [29]. The isolated *E. coli* were not serotyped in this study.

### Antimicrobial susceptibility testing

To study the resistance pattern of the isolates, the disk diffusion method on Mueller-Hinton agar with six antibacterial paper disks was used (HamoonTeb, Iran; Table 1). In summary, a number of pure bacterial colonies were suspended in tubes containing sterilized 0.9% saline in order to make its opacity equal to the 0.5 McFarland standard tube. They were then cultured on Mueller-Hinton agar. The antibacterial paper disks were placed on the agar and after 24 h of incubation at 37 °C, the diameter of the zone of inhibition was measured. The results were interpreted based on the Clinical and Laboratory Standards Institute guidelines [30].

### DNA extraction and detection of *tet* genes

The genomes of the isolated bacteria were extracted through boiling [31], using a thermomixer (Eppendorf, Germany), at 95 °C. In this study we investigated just the DNA genome, and the extracted DNA was stored at − 20 °C in 200 μl tubes until further analysis. Specific primers were used in order to identify the *tet*A, *tet*B, *tet*C, and *tet*D genes (Pishgam, Iran; Table 3). The Multiplex-polymerase chain reaction (PCR) mixture, with a final volume of 25 μl, contained 12 μl of 2X Master Mix (2X PCR Master Mix Red; Pishgam, Iran), 3 μl of DNA, 1 μl of forward primers (0.5 μl of *tet*A + 0.5 μl of *tet*D or 0.5 μl of *tet*B + 0.5 μl of *tet*C), 1 μl of reverse primers (0.5 μl of *tet*A + 0.5 μl of *tet*D or 0.5 μl of *tet*B + 0.5 μl of *tet*C), and 8 μl of sterilized distilled water. *E. coli* ATCC 25922 was used as a control strain [32]. The PCR reaction was performed using a thermocycler (Eppendorf, Germany) under the following program: one cycle of initial denaturation at 94 °C for 5 min and 35 cycles for the other steps including a denaturation step at 94 °C for 30 s, annealing of primers at 55 °C for 30 s, and an extension step at 72 °C for 30 s, followed by one cycle of final extension at 72 °C for 5 min [12]. The PCR products were electrophoresed on a 1% agarose gel and then studied in a Gel Doc Machine (Cambridge, Germany) after 20 min of exposure to ethidium bromide (CinnaGen, Iran).

**Table 3:** The primers used for detecting *tet*A, *tet*B, *tet*C and *tet*D genes in *Escherichia coli* isolates [12]

Target gene	Primer	Primer sequences (5′ to 3′)	Amplicon size (bp)
***tet*A**	*tet*A-F	CGCCTTTCCTTTGGGTTCTCTATATC	182
	*tet*A-R	CAGCCCACCGAGCACAGG	
***tet*B**	*tet*B-F	GCCAGTCTTGCCAACGTTAT	975
	*tet*B-R	ATAACACCGGTTGCATTGGT	
***tet*C**	*tet*C-F	TTCAACCCAGTCAGCTCCTT	560
	*tet*C-R	GGGAGGCAGACAAGGTATAGG	
***tet*D**	*tet*D-F	GAGCGTACCGCCTGGTTC	780
	*tet*D-R	TCTGATCAGCAGACAGATTGC	

### Statistical analysis

The data were analyzed statistically using SPSS® (version 20) software by Chi-square and Fisher’s exact tests. Differences were considered to be statistically significant at *p* < 0.05.

## Supplementary information

**Additional file 1.** Fig. 1 The original full-length gel image of the amplified products is presented.

## Data Availability

The data used and/or analyzed during the current study are available from the corresponding author on reasonable request.

## Abbreviations

*E. coli*: *Escherichia coli*
TSB: Tryptic soy broth
EMB: Eosin methylene blue
CLSI: Clinical and laboratory standards institute
ml: Milliliter
PCR: Polymerase chain reaction
